# Oncolytic Foamy Virus: Generation and Properties of a Nonpathogenic Replicating Retroviral Vector System That Targets Chronically Proliferating Cancer Cells

**DOI:** 10.1128/JVI.00015-21

**Published:** 2021-04-26

**Authors:** Karol M. Budzik, Rebecca A. Nace, Yasuhiro Ikeda, Stephen J. Russell

**Affiliations:** a Department of Molecular Medicine, Mayo Clinic, Rochester, Minnesota, USA; Ulm University Medical Center

**Keywords:** simian foamy virus, cancer, oncolytic virotherapy, targeting chronic proliferation, viral vectors

## Abstract

Nonpathogenic retroviruses of the *Spumaretrovirinae* subfamily can persist long term in the cytoplasm of infected cells, completing their life cycle only after the nuclear membrane dissolves at the time of cell division. Since the targeting of slowly dividing cancer cells remains an unmet need in oncolytic virotherapy, we constructed a replication-competent foamy virus vector (oFV) from the genomes of two chimpanzee simian foamy viruses (PAN1 and PAN2) and inserted a green fluorescent protein (GFP) transgene in place of the *bel-2* open reading frame. oFV-GFP infected and propagated with slow kinetics in multiple human tumor cell lines, inducing a syncytial cytopathic effect. Infection of growth-arrested MRC5 cells was not productive, but oFV genomes persisted in the cytoplasm, and the productive viral life cycle resumed when cell division was later restored. *In vivo*, the virus propagated extensively in intraperitoneal ovarian cancer xenografts, slowing tumor growth, significantly prolonging survival of the treated mice, and sustaining GFP transgene expression for at least 45 days. Our data indicate that oFV is a promising new replication-competent viral and gene delivery platform for efficient targeting of the most fundamental trait of cancer cells, their ability to sustain chronic proliferation.

**IMPORTANCE** The infectivity of certain retroviruses is limited to dividing cells, which makes them attractive tools for targeting cancer cell proliferation. Previously developed replication-competent gammaretroviral vectors spread efficiently in rapidly dividing cancer cells, but not in cancer cells that divide more slowly. In contrast to rapidly proliferating transplantable mouse tumors, slow proliferation is a hallmark of human cancers and may have contributed to the clinical failure of the preclinically promising murine leukemia virus vector Toca 511, which failed to show efficacy in a phase 3 clinical trial in patients with glioblastoma. The studies presented in our manuscript show that oFV vectors are capable of persisting unintegrated in quiescent cells and resuming their life cycle once the cells start dividing again. This property of oFVs, together with their lack of pathogenicity and their ability to catalyze the fusion of infected cancer cells, makes them an attractive platform for further investigation.

## Abstract (precis)

The infectivity of certain retroviruses is limited to dividing cells, which makes them attractive tools for targeting cancer cell proliferation. Previously developed replication-competent gammaretroviral vectors spread efficiently in rapidly dividing cancer cells, but not in cancer cells that divide more slowly.

## INTRODUCTION

Foamy viruses (FVs) are nonpathogenic, complex retroviruses estimated to have existed for over 400 million years (1). Their genomes are the largest among *Retroviridae* family members, reaching up to approximately 13 kb (simian foamy viruses [SFVs]) (2–4). FVs carry accessory genes *tas* and *bel2* located downstream of *env*. *tas* encodes the viral transcription *trans*-activator protein Tas, whereas a splicing event between *tas* and *bel2* leads to the creation of a new mRNA encoding the Bet protein, which has been shown to counteract APOBEC3G-apolipoprotein B mRNA editing enzyme, catalytic polypeptide-like 3G (5, 6), as well as playing a role in the viral latency process (7). The replication strategy of FVs is unusual, sharing features of both *Orthoretrovirinae* and *Hepadnaviridae* (8). After genome integration, FV gene expression is dependent on the activity of two viral promoters. The ubiquitously active internal promoter is located close to the 3′ end of the *env* gene and is crucial in the early phases of FV infection, as it drives the expression of the accessory proteins Tas and Bet (9). Accumulated Tas protein binds to the second FV promoter, located in the long terminal repeat (LTR), and activates the expression of the structural genes *gag*, *pol*, and *env* (9, 10). Expression from the LTR promoter is undetectable in the absence of the accessory Tas protein (11). No other retroviruses have yet been shown to carry an internal promoter (8, 12). FVs have a very broad tissue tropism, and different family members have evolved to infect a wide variety of mammals, including cats (feline foamy viruses), cattle (bovine foamy viruses), horses (equine foamy viruses), as well as monkeys and apes (simian foamy viruses). FVs are generally transmitted via saliva through biting and have been shown to replicate in oral mucosa (12, 13). SFVs are highly prevalent in nonhuman primates (NHPs), with infection rates between 44% and 100% (14); however, no SFV-induced pathology has been reported (13, 15). SFVs can replicate efficiently in human cells (16) and are capable of zoonotic transmission to humans occupationally exposed to nonhuman primates (15, 17). However, similar to the natural hosts of SFVs, infected humans do not develop signs of disease, and human-to-human transmission of SFVs has not been reported to date (15, 17–19).

The ability to sustain chronic proliferation is one of the most fundamental traits of cancer cells (20) and is often exploited to ensure the specificity of cancer therapies. Human tumors generally grow very slowly compared to preclinical mouse tumor models, with reported doubling times of their total mass varying from 7 to over 1,000 days, with medians varying from 34.5 to 511 days (21–25). Given that tumor mass doubling time is dependent on cell turnover, the rate of division of tumor cells is a key driver of this metric. In general, in comparison to cultured tumor cell lines, human tumor cells in intact tumors divide very slowly, with reported doubling times varying between 25 to over 200 days (26), compared to just 20 to 40 h for most lab-adapted cancer cell lines. This low rate of cell division likely limits the efficacy of S-phase-targeted therapeutic agents that cannot persist in the cancer cell and wait for cell division to occur.

Oncolytic virotherapy (OV) is an emerging modality of cancer therapy which utilizes viruses capable of selectively replicating in cancer cells to destroy tumors and activate antitumoral adaptive immune responses (27). The life cycles of many retroviruses are dependent on cell division (28), making them ideal candidates for targeting the unregulated proliferation of tumor cells. Replication-competent Moloney murine leukemia virus (MLV) oncolytic vectors were developed for cancer therapy and showed promise both in preclinical models and clinical trials (29–33). However, the MLV capsid has a very short intracytoplasmic half-life of 5.5 to 7.5 h (34) and cannot integrate its genome into the host cell chromosome until the nuclear membrane disintegrates during cell division, thus restricting its ability to infect and spread in slowly growing human tumors. This characteristic may have contributed to the recent failure of an oncolytic MLV (oMLV) in phase 3 clinical trials for glioblastoma (https://www.prnewswire.com/news-releases/tocagen-reports-results-of-toca-5-phase-3-trial-in-recurrent-brain-cancer-300916705.html).

Similar to MLV, a C-type retrovirus, FVs are S-phase dependent and preferentially infect dividing cells (35). However, in contrast to the case with C-type retroviruses, FV capsids have been shown to persist intact in the cytoplasm at the centrosome of quiescent cells for at least 30 days (36), which allows for successful resumption of the viral life cycle when the quiescent cells are stimulated to divide days or weeks after infection (37). We reasoned that this property should allow replicating FV vectors to infect slowly dividing tumor cells, resulting in more efficient spread of the virus in human tumors. FVs have additional characteristics which may be potentially attractive for cancer therapy, such as (i) their ability to kill infected cells via syncytium formation (16), (ii) their lack of pathogenicity, (iii) their significantly reduced potential to cause insertional mutagenesis due to their preferential insertion into chromosomal regions that do not contain genes and are not actively transcribed (38, 39), and (iv) the presence of an insulator sequence in the LTR which prevents activation of cellular genes by the FV enhancer (40, 41).

Here, we report the generation of recombinant chimeric chimpanzee simian foamy virus (SFVcpz) vectors engineered to incorporate foreign transgenes, here referred to as oncolytic FV (oFV). oFV efficiently infected multiple human cancer cell lines and propagated *in vivo* in intraperitoneal ovarian cancer xenografts, leading to tumor control and prolonged survival. In contrast to oncolytic MLV, in growth-arrested MRC5 cells, oFV preintegration complexes were able to latently persist for at least 64 h postinfection with preserved ability to resume the viral life cycle when the cells were stimulated to divide, thereafter proceeding to proviral integration and the production of viral progeny. oFV offers a promising new replication-competent retroviral gene delivery platform that targets both rapidly and slowly proliferating cells and therefore has potential as a candidate for a useful cancer therapy.

## RESULTS

### Generation of an oFV infectious molecular clone.

SFVcpzs replicate well in human cells (16) and are capable of infecting humans (4, 13); therefore, we chose two SFVcpz strains for our studies, PAN1 (SFV type 6) and PAN2 (SFV type 7) whose *env* nucleotide sequences exhibit 88.7% homology, indicating a close evolutionary relationship.

**FIG 1:**
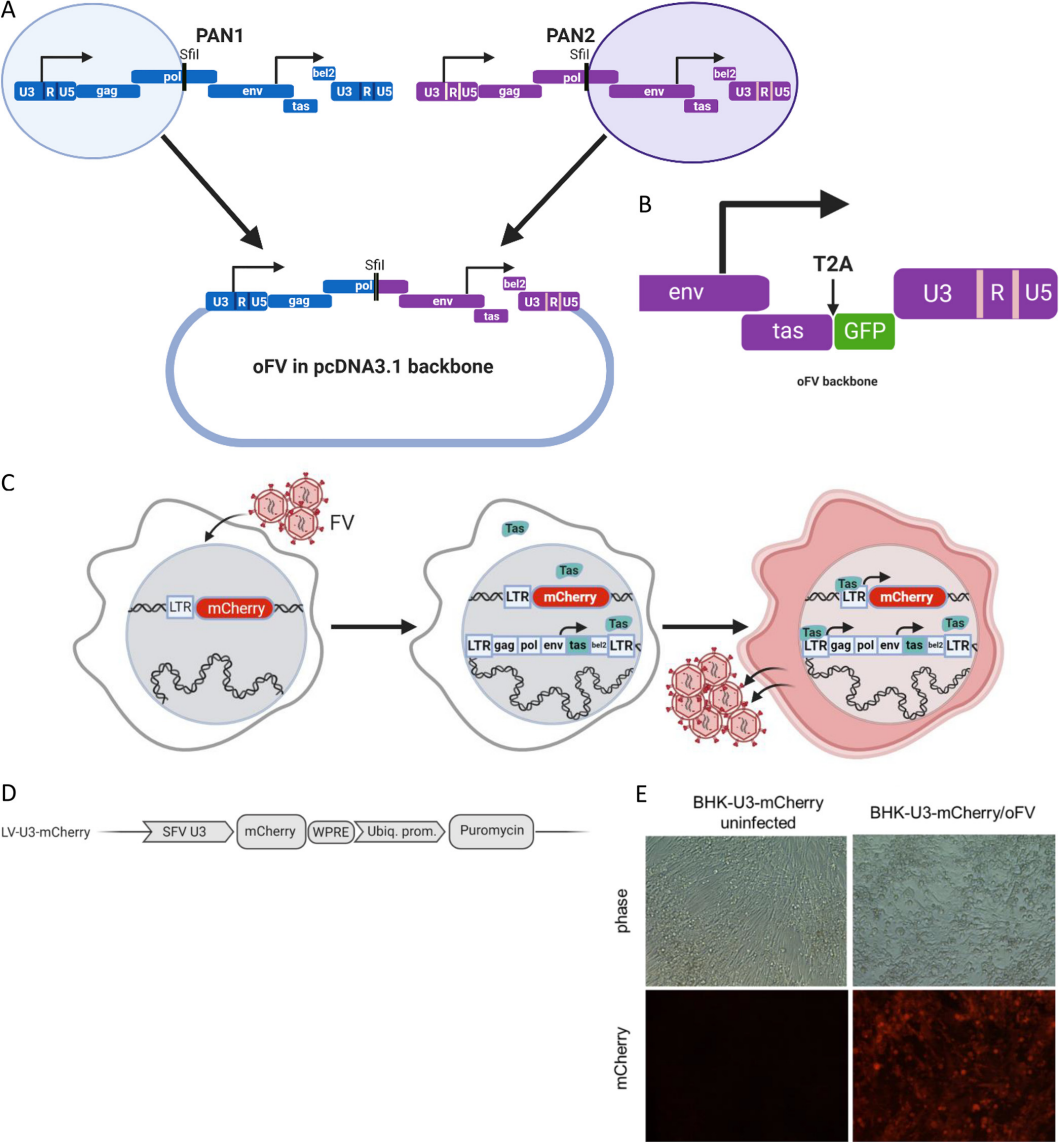
Generation of the oFV vectors and indicator cell lines for monitoring FV replication. (A) Infectious molecular clone for the chimeric oFV virus comprised of genome segments from PAN1 and PAN2 that were cloned into pcDNA3.1 and ligated together. (B) Engineering of the GFP-carrying virus. A part of *bel2* was replaced with emerald GFP cDNA and linked to *tas* via a T2A self-cleaving peptide. (C) Indicator cells contain a reporter gene driven by the U3 SFV promoter, which is activated by Tas during FV infection (see text for details). (D) Structure of the lentiviral vector used for generation of indicator cell lines. SFV U3, promoter-containing U3 region of the SFV LTR; Ubiq. prom., ubiquitin promoter; PGK prom, phosphoglycerate kinase promoter; puromycin, puromycin resistance cassette; neomycin, neomycin resistance cassette; WPRE, woodchuck hepatitis virus posttranscriptional regulatory element. (E) Indicator BHK-U3-mCherry cells express mCherry only when infected with oFV.

Infectious molecular clones were generated by combining the proviral genomes of PAN1 and PAN2. The molecular clone that gave rise to a virus of the highest fitness was a PAN1/PAN2 chimera, which was subsequently named oncolytic FV (oFV) (Fig. 1A). The first half of the oFV genome (from the 5′ LTR to the SfiI restriction site within *pol*) was copied from the PAN1 genome, whereas the remainder was derived from the PAN2 genome (Fig. 1A). In the absence of a foreign transgene payload, the length of the oFV proviral genome is 12,680 bp. To engineer a reporter gene into the oFV genome, we deleted a part of *bel2* between the end of *tas* and the polypurine tract and replaced it with a cDNA encoding the emerald green fluorescent protein (GFP) (Fig. 1B). To ensure translation of the eGFP transgene, we linked it to *tas* via a T2A self-cleaving connector peptide (Fig. 1B). The inserted sequence encoding the T2A-GFP translational unit was flanked by SacII and BspEII restriction sites. The length of the GFP-encoding proviral genome is 13,006 bp.

### Generation of BHK-U3-mCherry SFV indicator cell line for SFV titration.

To create a system allowing for easy and noninvasive oFV titration and monitoring of oFV replication *in vitro* and *in vivo*, we adopted a previously described strategy that exploits the LTR-transactivating activity of the virally encoded Tas protein (42, 43). Since the LTR promoter remains dormant until activated by Tas, we generated indicator cells stably transduced with reporter genes under the control of an inducible promoter corresponding to the U3 region of the FV LTR. Upon oFV infection and integration in these reporter cells, the FV provirus starts expressing Tas, which then activates not just the viral LTR but also the reporter gene (Fig. 1C). To engineer the indicator cell lines, we generated a lentiviral vector encoding mCherry driven by the U3 region of PAN1 (Fig. 1D) and used it to transduce BHK-21 and human glioblastoma U251 cells. The indicator BHK-21-U3-mCherry cells expressed mCherry only when infected with oFV (Fig. 1E) and were subsequently used for titration of all oFV vectors and viral growth dynamics studies described in the manuscript.

### Transgene insertion attenuates the oFV vector, but the GFP transgene is stable for up to 5 passages.

**FIG 2:**
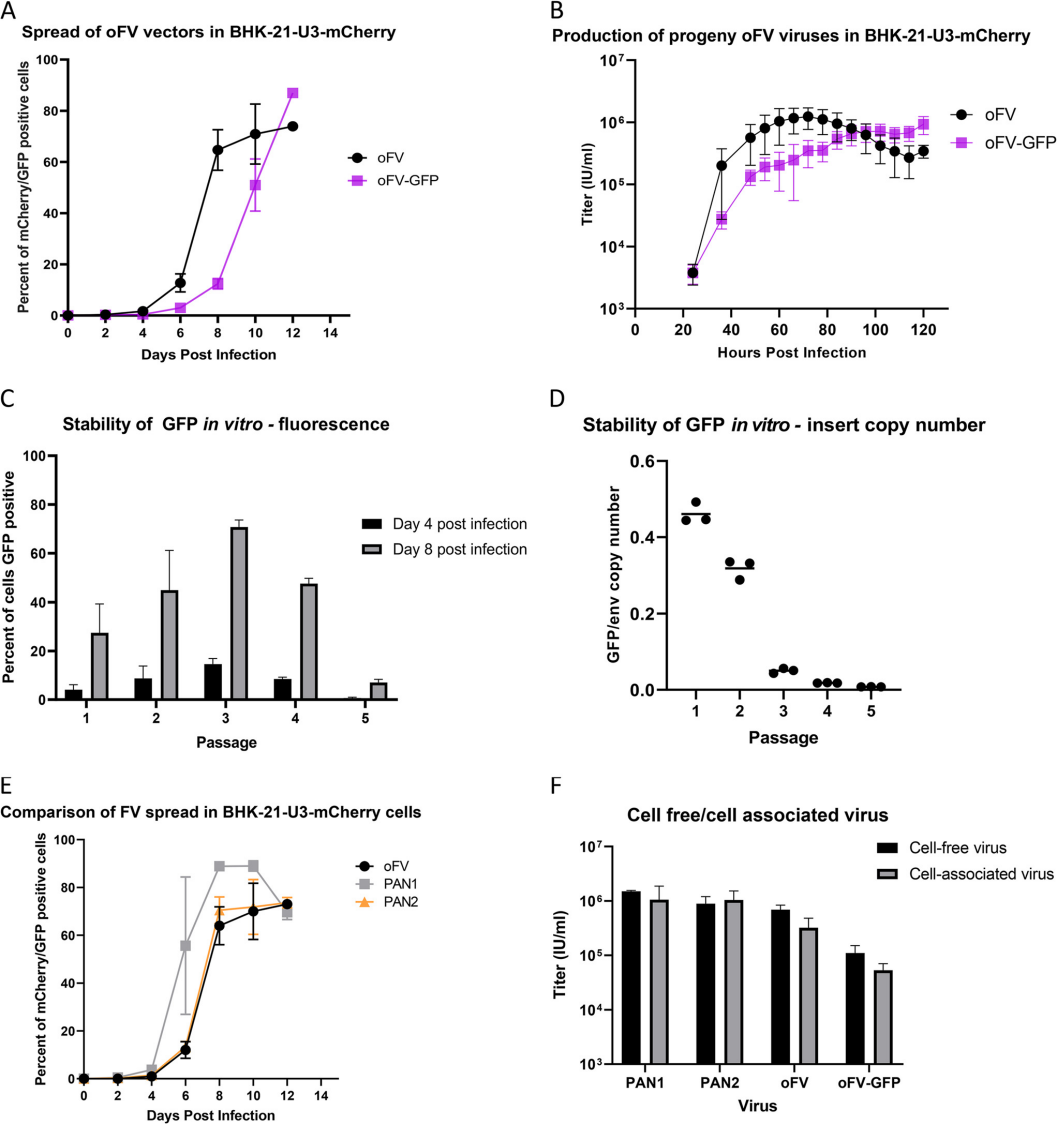
Transgene insertion attenuates the oFV vector, but the GFP transgene is stable for up to 5 passages. (A) Indicator BHK-U3-mCherry cells were infected at an MOI of 0.01 with oFV or oFV-GFP, and the spread of the viruses was assessed by sampling the cells every other day using flow cytometry analysis to determine the percentage of mCherry- or GFP-positive cells at each time point. Results of two independent experiments, each with two biological replicates, are presented. (B) BHK-U3-mCherry cells were infected at an MOI of 3 with oFV or oFV-GFP, and the titers of progeny virus were measured at different time points postinfection. Results of two independent experiments, each with two biological replicates, are presented. (C) oFV-GFP was passaged in U251-U3-mCherry cells 5 times. The first passage was done at an MOI of 0.5. For every subsequent passage, 250 μl of media from the previous passage was used for infection of fresh U251-U3-mCherry cells. Four and 8 days into each passage, the infected cells were analyzed by flow cytometry to determine the percentage of GFP-positive cells. Presented are results of 2 independent experiments with 1 biological replicate. (D) oFV-GFP was passaged in U251-U3-mCherry cells 5 times as described above. At the end of each passage, the infected cells were collected, their genomic DNA isolated, and *env* and GFP copy number relative to β-actin was determined using qPCR. Result of an experiment with 3 biological replicates is shown as GFP-to-*env* copy number ratio. (E) Indicator BHK-U3-mCherry cells were infected at an MOI of 0.01 with oFV or the parental PAN1 and PAN2 viruses, and their spread was assessed by sampling the cells every other day using flow cytometry analysis to determine the percentage of mCherry- or GFP-positive cells at each time point. Result of two independent experiments, each with two biological replicates, are presented. (F) Titers of cell-free and cell-associated progeny PNA1, PAN2, oFV, and oFV-GFP virions measured on day 8 postinfection at an MOI of 0.01. Results of two independent experiments, each with two biological replicates, are presented.

Multistep (Fig. 2A) and one-step (Fig. 2B) growth curves of parental and transgene-carrying oFV vectors were determined on BHK-21-U3-mCherry indicator cells. oFV and oFV-GFP exhibited slow spread in these cells, with a prolonged lag phase in growth lasting 4 (oFV) or 6 (oFV-GFP) days (Fig. 2A). The one-step growth curve revealed that progeny oFV titers peaked between 54 and 84 h postinfection at ∼1.5 × 10^6^ infectious units (IU)/ml (approximately 3 progeny virions per cell) (Fig. 2B). The release of progeny oFV-GFP was slower than for the parental virus, with supernatant titers peaking between 102 and 120 h postinfection at ∼8 × 10^5^ IU/ml. The slower spread and lower progeny yield of oFV-GFP than oFV indicated that transgene insertion can attenuate oFV.

We next investigated the stability of transgenes in the oFV backbone during serial passage. oFV-GFP was serially passaged five times on U251-U3-mCherry cells (Fig. 2C). For the first passage, the cells were infected at a multiplicity of infection (MOI) of 0.5; for every subsequent passage, 250 μl of the filtered progeny-containing media from a previous passage was used to infect U251-U3-mCherry cells. Each passage lasted 8 days, and GFP expression was determined by flow cytometry on days 4 and 8 of each passage. The percentage of GFP-positive cells relative to total oFV-infected cells decreased with each passage, falling to only ∼7% on day 8 of passage 5 (Fig. 2C). To further investigate the transgene loss during serial virus passage, we isolated the total DNA from the infected cells at the end of each 8-day passage and quantified the copy number of the GFP open reading frames as well as the FV *env* gene relative to the β-actin gene using quantitative PCR (qPCR) and calculated the GFP-to-*env* copy number ratio (Fig. 2D). This experiment revealed gradual transgene copy number loss over time, confirming our GFP expression analysis.

We also compared the parental PAN1 and PAN2 SFVcpz viruses with the oFV vectors in terms of *in vitro* spread and progeny production (Fig. 2E and F). In indicator BHK-U3-mCherry cells, oFV spread at kinetics very similar to PAN2, while PAN1 showed slightly faster kinetics (Fig. 2E). Replication of PAN1, PAN2, and oFV in BHK-21-U3-mCherry cells led to production of similar amounts of progeny, while the titers of oFV-GFP progeny were approximately 10-fold lower (Fig. 2F). Interestingly, the proportion of cell-free to cell-associated progeny virions was similar for all the viruses (Fig. 2F).

### oFV and oFV-GFP cause intercellular fusion, have a broad cancer tropism, and show potent oncolytic activity *in vivo*.

**FIG 3:**
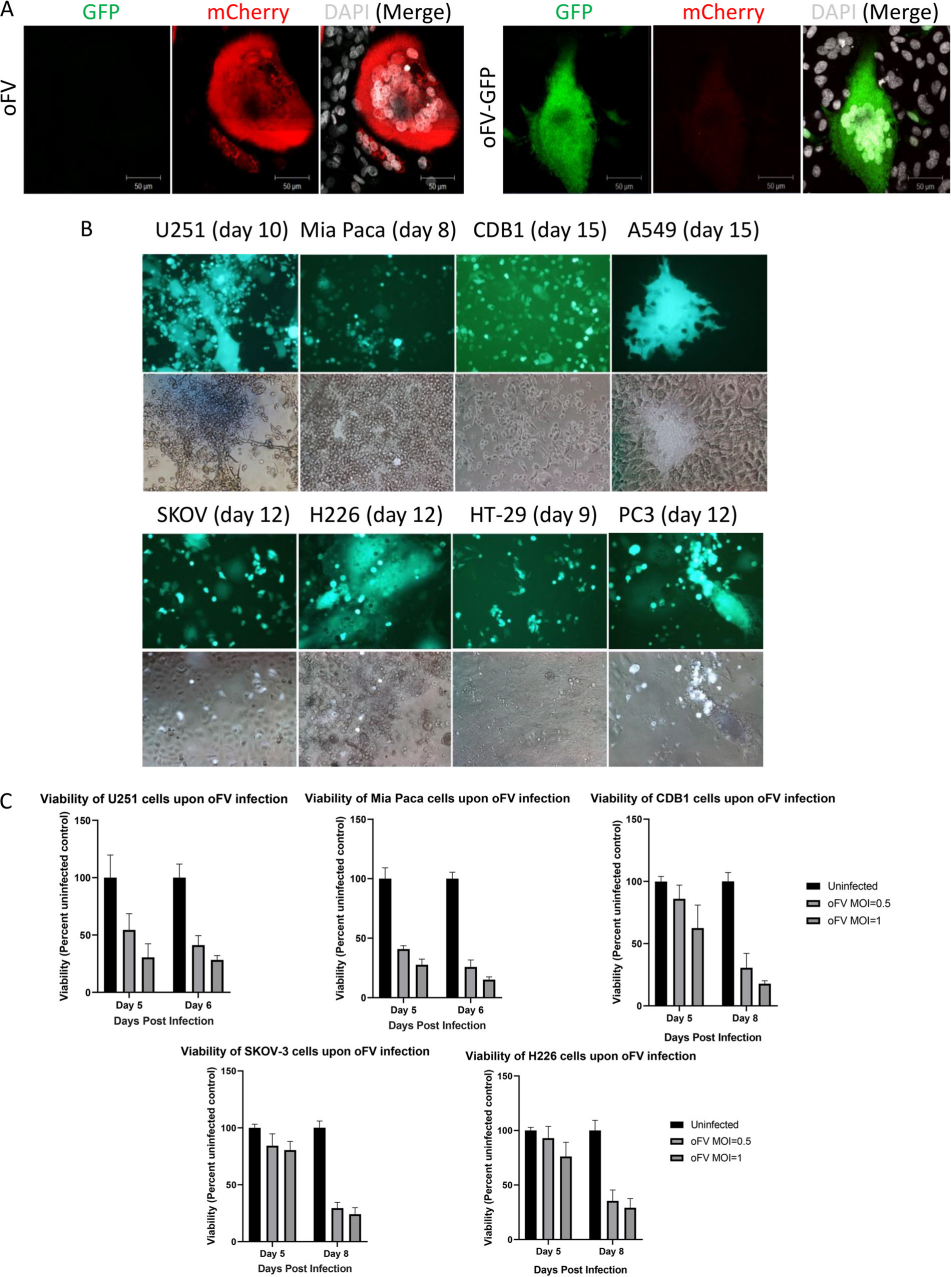
oFV and oFV-GFP cause intercellular fusion and have a broad cancer tropism. (A) Indicator U251-U3-mCherry cells were infected with oFV or oFV-GFP, and the syncytia induced by the virus were imaged with confocal microscopy (white, 4′,6-diamidino-2-phenylindole [DAPI]; red, mCherry; green, GFP). (B) Various human cancer cell lines were infected with oFV-GFP at an MOI of 0.5 and imaged for the expression of GFP on indicated days postinfection. U251, glioblastoma; Mia Paca, pancreatic adenocarcinoma; CDB1, cholangiocarcinoma; A549, lung adenocarcinoma; SKOV, ovarian carcinoma; H226, mesothelioma; HT-29, colorectal carcinoma; PC3, prostate adenocarcinoma. Extent of syncytium formation varies between different cell lines, with U251, H226, A549, and PC3 fusing well. (C) Various human cancer cell lines were infected with oFV at an MOI of 0.5 or 1, and viability was measured 5 and 6 or 8 days postinfection. Results of 2 independent experiments with 3 biological replicates are shown.

As expected based on observations of other FVs, infection with oFV or oFV-GFP led to syncytial cytopathic effect *in vitro*, as shown on U251-U3-mCherry cells in Fig. 3A. Similar syncytial cytopathic effects were observed in human cancer cell lines of diverse tissue origins, including pancreatic (MIA PaCa), glioblastoma (U251), ovarian (SKOV-3), cholangiocarcinoma (CDB1), and mesothelioma (H226) cells (Fig. 3B and C). The extent of syncytium formation varied between cell lines, with U251 and H226 fusing extensively, while HT-29, CDB1, and SKOV-3 fused less efficiently. Subsequent to the induction of syncytia, oFV infection resulted in a gradual loss of cell viability, leading to wholesale destruction and loss of monolayer viability by day 8 postinfection (Fig. 3C).

**FIG 4:**
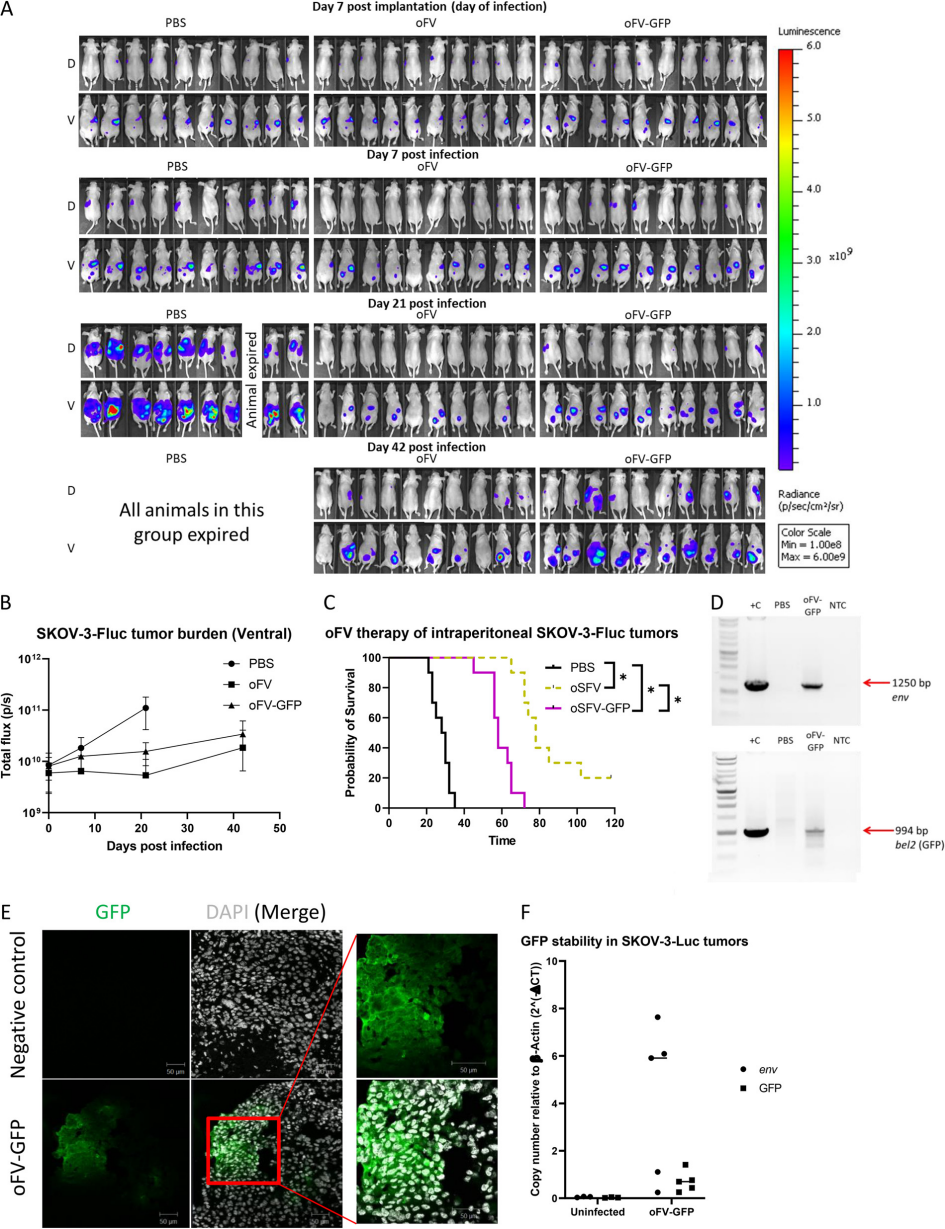
oFV and oFV-GFP have an oncolytic activity *in vivo*, and infection leads to intratumoral transgene expression. (A) We implanted 2.5 million SKOV-3-Fluc cells intraperitoneally in athymic nude mice and treated them with a single, intraperitoneal dose of 1 × 10^7^ IU of oFV, oFV-GFP, or PBS control 7 days post-tumor implantation. The tumor burden was measured at indicated time points by bioluminescence imaging with IVIS Lumina; 10 mice per group were used. Both oFV and oFV-GFP exhibited significant oncolytic potential *in vivo*. D, dorsal view; V, ventral view. (B) Quantification of the ventral bioluminescence signal in panel A. (C) Survival of the SKOV-3-luc-tumor-bearing mice after oFV vector therapy; *, *P* < 0.0001. (D) Tumors from PBS- and oFV-GFP treated mice which eventually succumbed to the disease were explanted. Total DNA was isolated from the explants and PCR analyzed using primers that bind within the oFV env gene and primers that bind upstream and downstream of the transgene insertion site within the remaining sequences of *bel2*. +C, positive control (poFV-GFP plasmid); NTC, no-template control. (E) Immunohistochemistry staining for GFP in sections of an oFV-GFP-infected tumor harvested 45 days postinfection. (F) Total DNA was isolated from oFV-GFP-infected tumors harvested from mice that succumbed to the disease on day 45 (tumor 1), 56 (tumors 2, 3, and 4), 65 (tumor 5), or 72 (tumor 6) postinfection, and GFP and env copy number relative to β-actin were measured using qPCR.

We next evaluated the oncolytic potential of oFV and oFV-GFP *in vivo* in a xenograft, orthotopic model of ovarian cancer intraperitoneal metastases. Seven days postintraperitoneal implantation of 2.5 million SKOV-3-Fluc cells stably expressing firefly luciferase for noninvasive evaluation of tumor burden, the mice were treated with a single intraperitoneal dose of 1 × 10^7^ IU of oFV, oFV-GFP, or phosphate-buffered saline (PBS) control. Infection with either virus slowed tumor progression in the treated mice (Fig. 4A and B), resulting in at least a doubling of median survival (Fig. 4C) relative to PBS-treated controls, with oFV leading to complete disappearance of the bioluminescence signal in some of the mice by day 21 postinfection (Fig. 4A). Tumors from a PBS-treated mouse (euthanized 23 days post-PBS treatment due to ascites formation) and an oFV-GFP-treated mouse (euthanized 45 days postinfection due to ascites formation) were harvested and divided into 2 parts: half was explanted, and half was embedded in optimal cutting temperature compound and then snap-frozen for subsequent cryosectioning and immunohistochemical analysis. Genomic DNA was isolated from fresh tissue and PCR analyzed for the presence of the oFV vector provirus (using primers binding within the oFV *env* gene) as well as the presence of the GFP transgene (using primers that bind upstream and downstream of the transgene insertion site within the retained sequences of *bel2*). Both the oFV provirus and the transgene were detected only in the oFV-GFP-infected tumor (Fig. 4D). Expression of GFP in the tumor was confirmed by immunohistochemical staining of sections of the snap-frozen part of the tumor (Fig. 4E). We then investigated the stability of the GFP transgene in the oFV backbone upon intratumoral amplification and spread. We collected tumors from mice treated with oFV-GFP that succumbed to the disease on days 45 to 72 posttreatment, isolated total DNA from the fresh tumor tissue, and quantified the copy number of the GFP open reading frame and the *env* gene relative to the β-actin gene using qPCR. This analysis revealed a substantially lower number of GFP copies relative to *env* in three of the analyzed tumors, suggesting partial loss of the transgene in those tumors (Fig. 4F). These data indicated that oFV and oFV-GFP have clear oncolytic activity *in vivo*, somewhat less so for oFV-GFP, but, in both cases, leading to significant prolongation of survival (Fig. 4C), in line with our observations from the *in vitro* studies (Fig. 2). Thus, despite its modest attenuation, oFV-GFP was capable of propagating and delivering its transgene *in vivo* and facilitating intratumoral expression of GFP; however, partial transgene loss was observed in some tumors.

### oFV-GFP genomes persist cytoplasmically in quiescent cells, and the virus therefore propagates faster than MLV-GFP in slowly dividing cancer cells.

**FIG 5:**
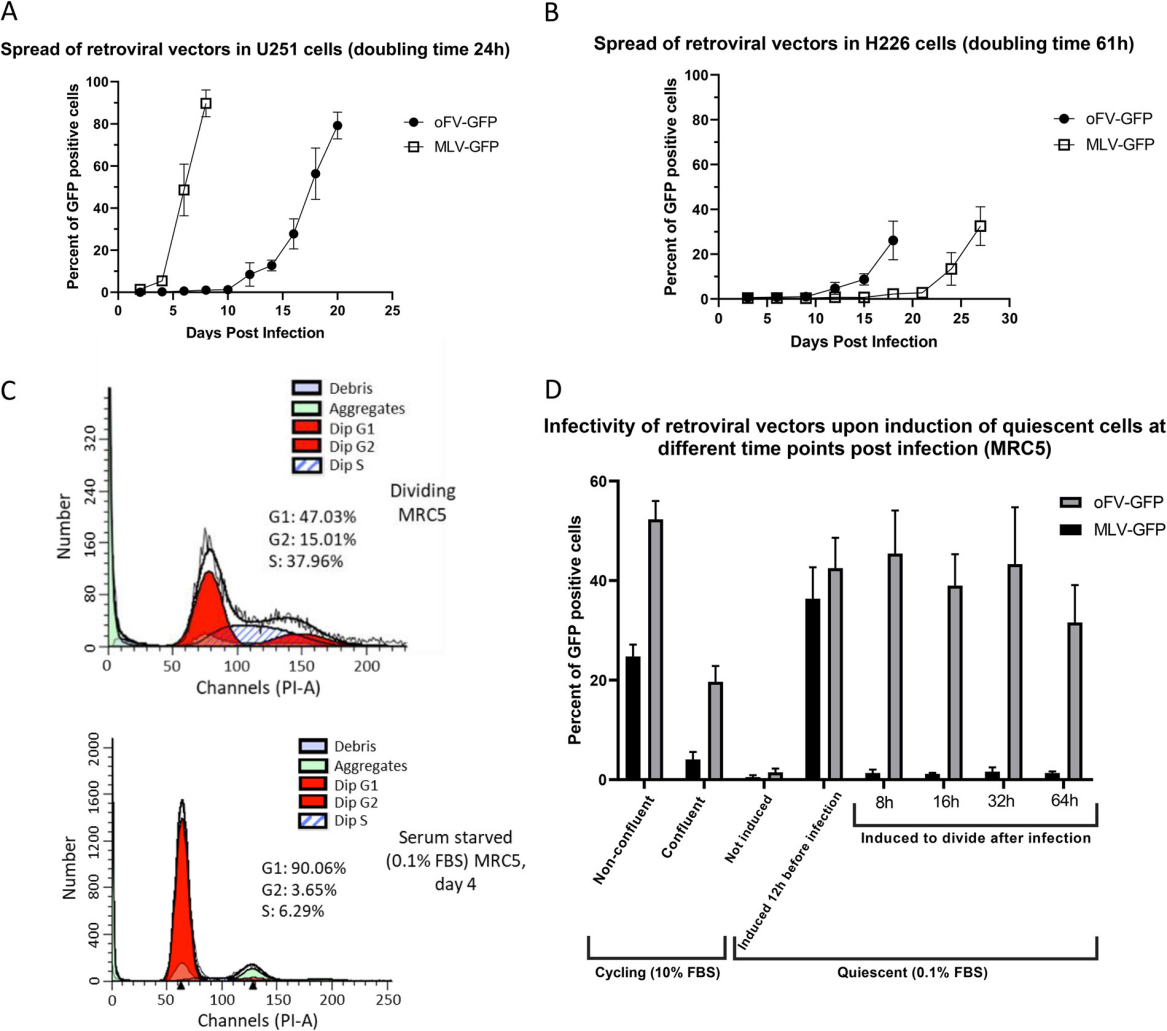
oFV-GFP spreads in slowly dividing cancer cells faster than MLV-GFP and, unlike MLV-GFP, can latently persist in quiescent cells. (A and B) Multistep growth curves of MLV-GFP and oFV-GFP in rapidly dividing U251 (A) and slowly dividing H226 (B) cancer cell lines; cells were infected at an MOI of 0.01, and the spread of the virus was followed by sampling the infected cells every 2 (U251) or every 3 (H226) days. Quantification of GFP-positive cells with flow cytometry; results of 2 independent experiments with 2 biological replicates are presented. (C) MRC5 cells were serum starved (0.1% FBS) for 4 days or cultured in 10% FBS media and stained with propidium iodide to determine the percentage of cells in different phases of the cell cycle and confirm the quiescent state of the cells. Analysis of the proportion of cells in different phases of the cell cycle was performed with ModFit LT V3.3.11. (D) Comparison of the ability of MLV-GFP and oFV-GFP to productively infect cells that are cycling, quiescent, or quiescent and induced to divide 12 h before infection or 8, 16, 32, or 64 h postinfection. All groups were infected with MLV-GFP or oFV-GFP at an MOI of 1. Results of 2 independent experiments with 3 biological replicates are shown.

To evaluate differences between oFV vectors and C-type retroviral vectors derived from Moloney murine leukemia virus (MLV), we compared oFV-GFP with the well-studied replication-competent MLV-GFP vector (44). As shown in Fig. 5A, multistep growth curves (MOI, 0.01) in rapidly dividing human glioblastoma U251 cells (doubling time, ∼24 h; https://web.expasy.org/cellosaurus/CVCL_0021) show that MLV-GFP spreads considerably faster than oFV-GFP, taking only half as long to infect 90% of the cells in the culture. However, in slowly dividing human mesothelioma H226 cells (reported doubling time, 61 h; https://web.expasy.org/cellosaurus/CVCL_1544), the picture is reversed, with MLV-GFP exhibiting greatly slowed replication compared to oFV-GFP (Fig. 5B). These above-mentioned findings support the hypothesis that oMLVs do not efficiently infect slowly cycling cells because their genomes have only a short half-life in the cytoplasm and that oFVs are able to overcome this limitation because their genomes can persist long term in the cytoplasm during prolonged G_0_. To further verify this hypothesis, we compared the infectivity of oFV-GFP and MLV-GFP in quiescent (serum-starved) MRC5 cells that were subsequently induced to complete the cell division cycle by adding serum at different time points postinfection. Quiescence in the MRC5 cells was induced by serum starvation for 5 days and was confirmed by propidium iodide staining (Fig. 5C). The quiescent cells were then infected with MLV-GFP or oFV-GFP (MOI, 1.0) and were later released from serum starvation 8, 16, 32, or 64 h postinfection (Fig. 5D). Neither virus productively infected serum-starved cells. However, when serum starvation was ended 12 h prior to infection, the productive infectivity of both viruses was fully restored. In cells that were released from serum starvation 8 h or later after infection, MLV-GFP did not replicate productively, but the productivity of the oFV-GFP infection was almost fully restored (Fig. 5D). These results confirm that, unlike MLV-GFP, oFV-GFP is able to latently persist in quiescent cells and resume its life cycle once the cells start dividing again.

### oFV-GFP exhibits stronger cytotoxicity and transgene expression than MLV-GFP.

**FIG 6:**
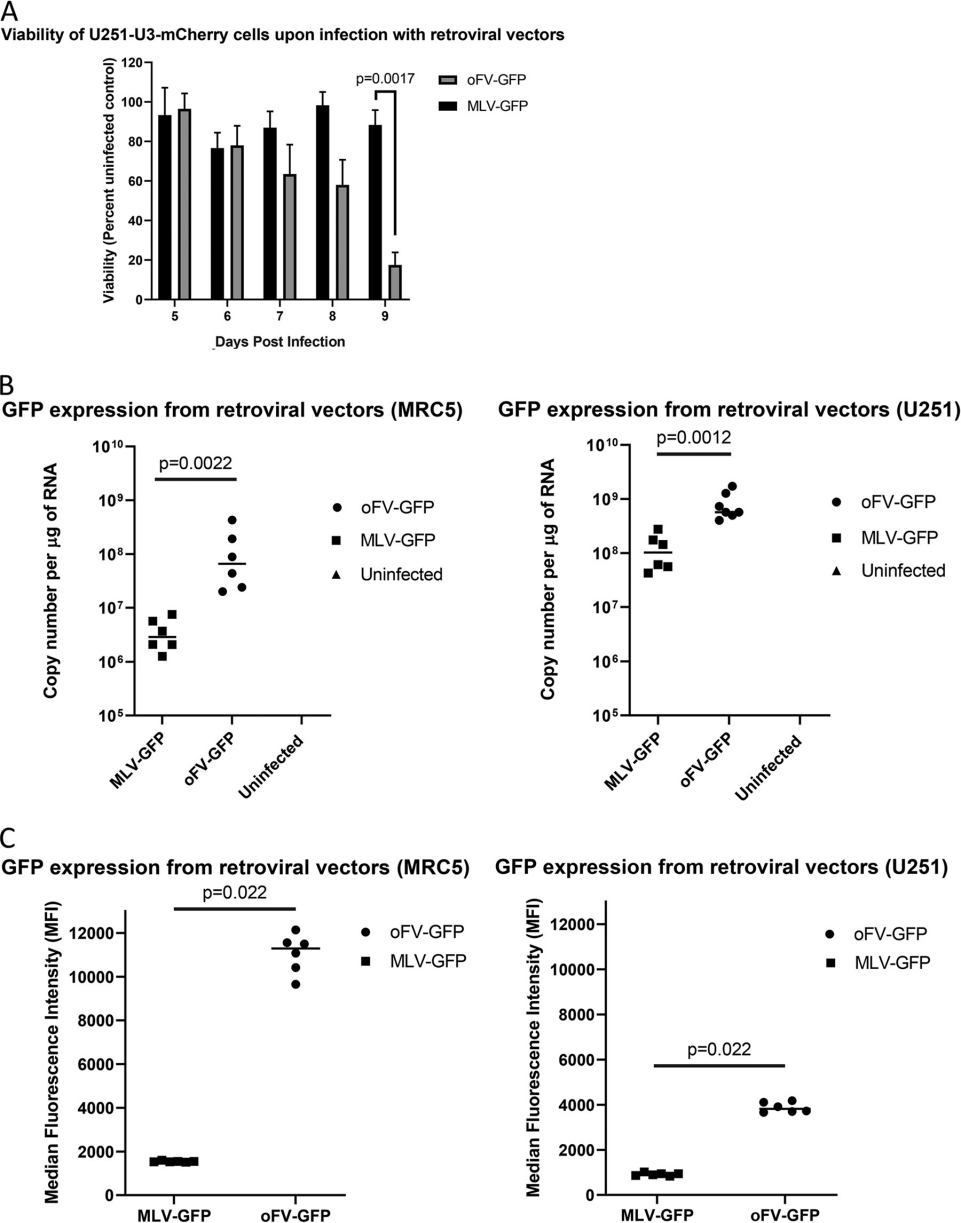
oFV-GFP exhibits stronger cytotoxicity and transgene expression that MLV-GFP. (A) Viability of U251 cells infected with MLV-GFP or oFV-GFP. Unlike MLV, oFV kills infected cells (statistical significance determined using the Holm-Sidak method; *n* = 3). (B)s Comparison of GFP expression in MRC5 and U251 cells infected with MLV-GFP and oFV-GFP 3 days postinfection at an MOI of 1 using qRT-PCR Statistical significance determined using the Mann-Whitney test; 2 independent experiments with 3 biological replicates. (C) Comparison of GFP expression in MRC5 and U251 cells infected with MLV-GFP and oFV-GFP 3 days postinfection at an MOI of 1 using flow cytometry. Statistical significance was determined using the Mann-Whitney test; *n* = 6.

We next compared the cytotoxicities and transgene expression profiles of oFV-GFP and MLV-GFP, using rapidly dividing U251 cells as the indicator line. In these cells, MLV-GFP did not cause cytotoxicity (Fig. 6A), whereas oFV-GFP infection led to intercellular fusion and loss of cell viability (Fig. 6A). Interestingly, regarding transgene expression, peak GFP mRNA copy number on day 3 postinfection was significantly higher in cells infected with oFV-GFP versus those infected with MLV-GFP, irrespective of the cell substrate and the rate of cell division (Fig. 6B). These data were corroborated by a flow cytometry analysis of GFP protein expression conducted 3 days post-oFV-GFP and MLV-GFP infection, revealing a significantly higher median fluorescence intensity following oFV-GFP infection than MLV-GFP infection, both in slowly and rapidly dividing cell lines (Fig. 6C).

## DISCUSSION

The promising efficacy data generated using Toca 511, a recombinant oncolytic Moloney MLV, in preclinical glioblastoma models (29, 30) suggested that replication-competent S-phase-targeted retroviral vectors might prove useful for cancer therapy. However, Toca 511 ultimately failed to show benefit in a phase 3 randomized clinical study, and published correlative studies from phase 1 clinical trials point to relatively low efficiency of tumor cell transduction in glioblastoma patients treated with intratumoral Toca 511 (45). Retroviral vectors derived from Moloney MLV have been shown to be unstable in the cytoplasmic compartment of infected cells, and they decay intracellularly, with a half-life of 5.5 to 7.5 h (34). As a result, their ability to successfully integrate their genomes and productively infect slowly dividing cells is largely impaired. Due to this intracellular instability of the viral nucleocapsid, the spread of the Toca 511 virus is expected to be quite inefficient in human tumors due to their low rate of growth and cell turnover relative to mouse tumors derived from rapidly cycling cell lines.

Here, in an attempt to develop an oncolytic retroviral vector capable of efficiently transducing slowly growing tumors, we generated oFV and demonstrated that it is indeed capable of infecting slowly cycling tumor cells. oFV-GFP, unlike MLV-GFP, was able to persist for at least several days in infected quiescent cells and to resume its life cycle once the cells were stimulated to divide up to 64 h postinfection, in line with previous studies of foamy virus biology (36, 37, 46). FV capsids have been shown to accumulate at the centrosome after entry into resting cells, where they remain intact for several weeks, which physically protects the viral genome from degradation (36). Once the cells are stimulated to divide, Gag proteolysis and genome uncoating occur, allowing the preintegration complex to access host cell chromosomal DNA, leading to proviral integration and resumption of the productive viral life cycle (36). oFV-GFP was also able to spread more efficiently than MLV-GFP in slowly dividing human mesothelioma H226 tumor cells, indicating that the MLV-based OV system is indeed hindered by its inability to productively infect cells that are not actively dividing at the time of viral entry.

Previously described replication-competent FV vector systems were derived from feline and prototype FVs. Feline foamy virus (FFV) vectors were constructed by inserting a GFP transgene downstream of *bel2*, either as a Bet-GFP fusion or translated separately from an internal ribosomal entry site (IRES), and were shown to drive expression of functional GFP in cat cells (47). Recently, an FFV vector expressing the feline immunodeficiency virus (FIV) Vif protein in place of a fragment of *bel2* was investigated as a potential FIV vaccine for cats (48). However, due to the inability of FFV to productively infect humans (12, 49), FFV vectors are considered unsuitable for human virotherapy applications. Prototype foamy virus (PFV), isolated from a Kenyan patient but later shown to be closely related to SFVcpz type 6 (50), was engineered to encode a foreign transgene by Axel Rethwilm and colleagues at the University of Würzburg. However, there have been no additional reports on this system since an initial promising publication in 2005 in which PFVs engineered to carry suicide genes were shown to be minimally effective in the treatment of subcutaneous human glioma tumors in nude mice (51).

Compared to MLV-GFP, oFV-GFP propagated slowly in rapidly cycling U251 cells *in vitro*. The slowness of the oFV replication cycle relative to that of MLV is likely a consequence of the lag time required before Tas-dependent transcription from the viral LTR ramps up. Expression of the reporter, which is driven by the Tas-dependent LTR promoter, was first detected 30 to 40 h postinfection (data not shown).

*In vivo*, in the intraperitoneal xenograft SKOV-3-Fluc ovarian cancer model, a single intraperitoneal injection of oFV and oFV-GFP potently controlled tumor growth, possibly due to the fusogenic properties of the oFV Env protein. Viruses that are naturally fusogenic or have been engineered to encode fusogenic glycoproteins have been shown to have anticancer properties, both in preclinical models (52, 53) and in clinical studies (54). Syncytia induced by fusogenic viral glycoproteins are short-lived and can grow by fusing with neighboring cells, allowing a single virus particle to kill more than one cell (55). The loss of viability by syncytia is associated with strong immunogenicity, as the dying syncytia release damage- and pathogen-associated molecular patterns, which, in the context of functional adaptive immune responses, can contribute to the antitumoral properties of fusogenic viruses by promoting cytotoxic T cell activity (55).

The oncolytic properties of oFV-GFP led to a significant prolongation of survival of the vector-treated mice; however, all the mice eventually developed ascites and were euthanized. This is possibly due to the emergence of a subpopulation of tumor cells that are resistant to the oncolytic properties of the virus, which facilitate chronic rather than lytic infection, leading to an equilibrium state between the virus and the tumor. A similar phenomenon in the context of oncolytic measles virus therapy has been described previously in the xenograft intraperitoneal ovarian cancer metastases model (56). This tumor model is associated with other limitations, such as (i) limited ability of oFV to spread in mouse cells (data not shown), which constricts the ability to fully assess the possible off-target toxicities associated with the oFV therapy; (ii) formation of subcutaneous tumors at the site of intraperitoneal tumor cell injection, which leads to premature euthanasia of mice without a substantial intraperitoneal tumor burden that could survive a longer period of time (this issue limited the survival of 4 of the parental oFV virus-treated mice); as well as (iii) the lack of functional adaptive immune responses in the tumor-bearing mice that could prevent a chronic intratumoral infection with the oFV vectors as well as possibly potentiate their antitumor properties.

In line with its potent antitumor activity, oFV-GFP propagated in tumors *in vivo* and delivered the GFP transgene to infected cells. The propagation likely occurred via both cell-associated (incorporation of uninfected cells into the infected syncytia) as well cell-free spread (release of progeny particles from viable syncytia and infection of neighboring cells). However, *in vitro* experiments showed that the GFP transgene was progressively disrupted over multiple rounds of amplification, suggesting that the inclusion of a foreign transcription unit in the virus may confer a selective disadvantage. Interestingly, it was previously reported that FV genomes are more prone to deletion upon *in vitro* versus *in vivo* virus amplification (57), which is in line with the greater stability of the oFV genome we observed after intratumoral virus propagation. We will further investigate the potential issue of stability in future studies by investigating oFV vectors containing transgene payloads of various sizes. This will also allow us to determine the largest transgene cargo size that oFV vectors can tolerate without compromising viral titers and the optimal positioning of transgene payloads to ensure stability of therapeutic cassettes over multiple rounds of replication. Furthermore, we will arm oFV with a variety of therapeutic transgenes encoding “suicide” proteins, cytokines, chemokines, checkpoint antibodies, and other immunomodulatory proteins, and we will evaluate whether these arming strategies can enhance the efficacy of oFV therapy. Efficacy evaluations will be extended to include both patient-derived xenograft (PDX) and immunocompetent, syngeneic mouse tumor models. PDX tumor models better reflect human cancer biology than cell line-based cancer models due to maintenance of key features of the donor tumor (58), including its slow and variable tumor growth kinetics (59), which will allow us to directly test whether oFV therapy truly is more efficacious than oMLV therapy in slow-growing tumors. Additionally, we will explore the use of integration-defective oFV vectors as an attempt to further increase the safety of this platform.

In summary, oFV is a chimeric S-phase-dependent retrovirus capable of infecting and spreading in slowly dividing cancer cells, thereby targeting their chronically proliferative phenotype, which is the primary hallmark of any cancer. Additional positive attributes of this new platform are the nonpathogenic nature of foamy viruses, a reduced risk of insertional mutagenesis compared to C-type retroviruses, the ability to kill infected cancer cells (via cell fusion), and the ability to drive high-level expression of a foreign transgene. oFV should be further developed as a replication-competent retroviral vector for cancer therapy.

## MATERIALS AND METHODS

### Cells, plasmids, and viruses.

293T (ATCC CRL-11268), U251 (kindly provided by Kah-Whye Peng, Mayo Clinic), BHK-21 (ATCC CCL-10), CDB1, A549 (ATCC CCL-185), and MIA PaCa-2 (ATCC CRL-1420) cells were cultured in Dulbecco’s modified Eagle’s medium (DMEM) supplemented with 10% fetal bovine serum (FBS) and 1% streptomycin/penicillin. SKOV-3 (ATCC HTB-77) and HT-29 (ATCC HTB-38) cells were cultured in McCoy's 5A (modified) medium supplemented with 10% FBS and 1% streptomycin/penicillin. Mesothelioma NCI-H226 (ATCC CRL-5826) and PC3 (ATCC CRL-1435) cells were cultured in RPM-1640 medium supplemented with 10% FBS and 1% streptomycin/penicillin. MRC5 (ATCC CCL-171) cells were cultured in Eagle’s minimal essential medium (EMEM) media supplemented with 10% (or 0.1% for serum starvation) FBS and 1% streptomycin/penicillin.

PAN1 (ATTC VR-632) and PAN2 (ATTC VR-633) viruses were obtained from ATCC and propagated in BHK-21 cells.

MLV-GFP plasmid (30) was kindly provided by Richard Vile, Mayo Clinic.

### Infectious clone construction.

The chimeric oFV infectious molecular clone (SFVcpz PAN1/PAN2 chimera) was generated by PCR amplification (*Ex Taq* polymerase; TaKaRa) of the PAN1 provirus genome from U3 of the 5′ LTR to 77 bp downstream of the unique restriction site SfiI localized in the *pol* gene (primers forward, ggccgcgcgctagcAAAGAAAGATGAGTATTATAG, and reverse, atatatagcggccgcTGTGAAGGCGGCAAAGGTCCAATA) and the genome of PAN2 from 98 bp upstream of the defective SfiI site (containing a point mutation in the SfiI site) to U5 of the 3′ LTR (primers forward, atatgctagcTGTGGTTAAGCAACTGGGACGTT, and reverse, gtatagcggccgcTTAAGATAAGTGTAGTTCAC); total DNA from U251 cells infected with PAN1 or PAN2 was used as the templates for the PCRs. Both fragments, ∼7 kb long each, were cloned into the expression vector pcDNA3.1(+) using NheI and NotI restriction sites. In order to recreate the functional SfiI restriction site of the PAN2 genome fragment, we used mutagenizing primers (forward, CTTCTGGCCCTATATTAAGGCCAGATAGGCCTCAAAAGC, and reverse, GCTTTTGAGGCCTATCTGGCCTTAATATAGGGCCAGAAG) replacing the mutated A base in the defective SfiI restriction site with the correct G base (LA *Taq* polymerase; TaKaRa). The corrected SfiI site was used to make a full-length infectious clone in the pcDNA3.1(+) backbone, generating the plasmid poFV.

To create a GFP-encoding virus, we used the gene synthesis services of GenScript. A segment of the oFV genome from the AgeI restriction site in the *tas* gene to the 3′ end of the genome (NotI restriction site) was synthesized, where a portion of the *bel2* open reading frame (ORF) (from the 3′ end of the *tas* gene to the polypurine tract) was replaced with the gene encoding emerald GFP. The *tas* and *gfp* ORFs were separated by a self-cleaving T2A sequence, enabling the translation of the *gfp* gene. The T2A-*gfp* expression cassette was flanked by BspEI and SacII restriction sites. The synthesized segment was then inserted into the poFV plasmid using the AgeI and NotI restriction sites.

The sequence of the poFV plasmid was verified by next-generation sequencing service of the Massachusetts General Hospital DNA core.

### Virus rescue.

To rescue the oFV viruses we transfected the constructed infectious clones into 293T cells using FuGENE 6 (Roche, Indianapolis, IN, USA) in a 10-cm dish. Two and 4 days posttransfection, the producer cells were split 1:2 and transferred to 15-cm dishes, and indicator BHK21-U3-mCherry cells were added to the culture. Six days posttransfection, the medium containing the viral supernatant was collected, and the intracellular viral particles were then released from the cells by 2 cycles of freezing and thawing. Finally, the virus prep was filtered through a 0.45-μm syringe filter (Millipore) and concentrated by ultracentrifugation at 50,000 × *g* for 2 h at 4°C in the SW 32 Ti rotor (Beckman) with a 20% sucrose cushion. The pellet was resuspended in PBS and stored at −80°C.

MLV-GFP was rescued by transfection of an MLV-GFP clone (30) (kindly provided by Richard Vile, Mayo Clinic) into 293T cells. Three days posttransfection, the medium containing the virus was collected, filtered through a 0.45-μm syringe filter, and concentrated with Retro-X concentrator (TaKaRa). The pellet was resuspended in PBS and stored at −80°C.

### Lentiviral vectors.

Second-generation lentiviral vectors (described previously [60]) were created for the generation of oFV indicator cells, using the following plasmids: pHR-SIN (vector), p8.91 QV (Gag-Pol expression construct), and pMD-G (vesicular stomatitis virus glycoprotein G [VSV-G] expression construct). The U3 region of PAN1 was PCR amplified (forward primer, ATAGAATTCGGAGAGGGTGTGGTGGAATG; reverse primer, CGAGGATCCTGAAGAGCTCTCGTACAA) and inserted as a promoter sequence upstream of the mCherry gene in a lentiviral vector backbone (pHR-SIN) carrying a puromycin resistance cassette. The lentiviral vectors were produced by transient transfection of 293T cells using FuGENE 6 (Roche) with a weight ratio of 2:1:1 of vector, Gag-Pol expression plasmid, and VSV-G expression plasmid. The vectors were harvested 72 h after transfection, filtered through a 0.45-μm filter membrane (Millipore), and either immediately used for transduction (of U251 or BHK-21) or stored at −80°C. Vector-transduced cells were selected with puromycin (3 to 7 μg/ml).

### Virus titer determination.

For titration of oFV vectors, we used 10^5^ BHK-21-U3-mCherry cells per well in a 24-well plate and infected them with 0.2, 1, or 5 μl (or 5, 10, or 50 μl for nonconcentrated virus) of virus prep. Seventy-two hours postinfection, the cells were harvested for a flow cytometry analysis to determine the percentage of mCherry-positive cells. For the comparison studies with MLV-GFP, oFV-GFP was titrated by infecting 10^5^ of MRC5 or U251 cells per well in a 24-well plate with 0.2, 1, or 5 μl of virus prep, and 72 h postinfection, the cells were collected for a flow cytometry analysis to quantify the GFP-positive cells.

MLV-GFP was titrated on 293T or MRC5 and U251 (for the oFV/MLV comparison experiments in MRC5 and U251) cells by infecting 10^5^ cells in a 24-well plate. Twenty-four hours postinfection, the cells were treated with azidothymidine (50 μM) and collected for flow cytometry analysis to quantify the number of GFP-positive cells 48 (293T) or 72 (MRC5) hours postinfection.

Viral titers were calculated by multiplying the number of cells seeded for infection by the fraction of mCherry- or GFP-positive cells and volume of viral prep used for infection in milliliters.

### Flow cytometry.

Cells were dispersed into a single-cell suspension by incubation in Versene (Gibco) at 37°C for 30 min, fixed in 4% paraformaldehyde (PFA), and analyzed using the LSR II flow cytometer (BD Biosciences). Results were analyzed using the FlowJo software.

### Virus growth dynamics analyses.

**(i) Multistep growth curves.** We seeded 1 × 10^5^ BHK-21-U3-mCherry or U251-U3-mCherry-U3-Luc or mesothelioma H226 cells in a well of a 6 or 12 (H226)-well plate. Three hours later, the cells were infected with indicated foamy viruses or MLV-GFP at an MOI of 0.01. Every 2 or 3 days (H226 cells), the cells were collected for a flow cytometry analysis of mCherry (or GFP)-positive cells. Infected cells were passaged at a 1:3 ratio every 4 or 6 days (H226). The analyses were done in duplicates in 3 independent experiments.

**(ii) One-step growth curve.** In a 48-well plate, 5 × 10^4^ BHK-U3-mCherry cells per well were seeded. Three hours later, the cells were infected with oFV or oFV-GFP at an MOI of 3. The supernatants were sampled (100 μl) 12, 24, 36, 48, 54, 60, 66, 72, 78, 84, 90, 96, 102, 108, 114, and 120 h postinfection, and the FV titers in those supernatants were measured using BHK-21-U3-mCherry cells. The analysis was done in duplicates in two independent experiments.

### Cell viability assays.

Two times 10^4^ target cells were seeded per well of a 96-well plate and infected with FVs and MOIs specified in the text. Five to nine days postinfection, the viability of the cells was measured using the PrestoBlue cell viability reagent (Invitrogen). Upon incubation with the reagent for 15 to 25 min, the fluorescence was read with Tecan Infinite M200 Pro at 560 nm (excitation) and 590 nm (emission). The results are shown as percentage of uninfected or untreated control.

### qRT-PCR and qPCR assays.

For the comparison of GFP expression by MLV-GFP and oFV-GFP, we first titrated both viruses on MRC5 or U251 cells. Then, 10^5^ MRC5 or U251 cells were infected with MLV-GFP or oFV-GFP at an MOI of 1, along with an uninfected control. Three days later, the cells were collected, and RNA was isolated using the RNeasy minikit (Qiagen). Reverse transcription-quantitative PCR (qRT-PCR) was performed on 50 ng of RNA with TaqMan RNA-to-CT 1-step kit (Applied Biosystems) using ViiA 7 real-time PCR system. The primers (forward, CCACATGAAGCAGCAGGACTT, and reverse, GGTGCGCTCCTGGACGTA) and probe (56-FAM/TTCAAGTCC/ZEN/GCCATGCCCGAA/3IABkFQ) used in this experiment have been previously described (61). GFP-encoding RNA of a known size was used as a copy number standard, diluted in cellular RNA isolated from MRC5 or U251 cells.

For the quantification of the GFP and *env* copy number, we performed qPCR on 50 ng of DNA isolated from oFV-GFP-infected (or uninfected controls) SKOV-3-Fluc tumors or U251 cells (total DNA isolation performed with DNeasy blood and tissue kits; Qiagen) with TaqMan universal PCR master mix (Applied Biosystems) and the ViiA 7 real-time PCR system. For GFP copy number quantification, we used the primers and the probe listed above. For the quantification of *env*, we used the following primers: forward, GTCACTCAGAGGGCTGTTTATC; reverse, CTTGTGGGATACTGGTCATGT; and probe,/56-FAM/CGTTCCCTT/ZEN/AGAGTGCAACACCCA/3IABkFQ/. The analysis was performed relative to the β-actin copy number using PrimeTime Std qPCR assay Hs.PT.56a.40703009.g, (Integrated DNA Technologies).

### Cell cycle inhibition assay.

MRC5 cells were cultured in 0.1% FBS EMEM or 10% FBS EMEM (cycling control) and were collected for propidium iodide staining 5 days after serum starvation had begun. The cells were then washed with PBS, fixed in 1% PFA, and washed twice. Subsequently, they were treated with 70% ethanol, incubated overnight at −20°C, washed, and stained with propidium iodide solution (20 μg/ml) containing RNase A (500 μg/ml). This was followed by a flow cytometry analysis to determine the proportion of cells in G_1_, S, and G_2_ phases of the cell cycle. The analysis was carried out with ModFit LT v3.3.11 software.

To compare the infectivity of MLV-GFP and oFV-GFP in quiescent cells, we cultured MRC5 cells in 0.1% EMEM for 5 days and then infected the cells with oFV-GFP or MLV-GFP at an MOI of 1 (viruses were previously titrated on cycling MRC5 cells). The cells were released from serum starvation 8, 16, 32, or 64 h postinfection, or 12 h before infection, by transferring from a 24- to a 12-well plate and culture in 10% EMEM. Three days postreactivation, the cells were collected for a flow cytometry analysis to quantify the percentage of GFP-positive cells. We also included the following controls: serum starved (not released from serum starvation and collected for flow cytometry 3 days postinfection) and cultured in 10% FBS EMEM confluent and nonconfluent (collected for flow cytometry 3 days postinfection).

### Transgene stability analysis.

We infected 10^5^ U251-U3-mCherry cells in a 6-well plate with oFV-GFP at an MOI of 0.5. Four days postinfection, the infected cells were passaged 1:3 and collected for a flow cytometry analysis. Eight days postinfection, the infected cells were collected for a flow cytometry analysis, while the supernatants were filtered through a 0.45-μm syringe filter (Millipore), and 0.25 ml of the filtered supernatant was used to infect 10^5^ U251-U3-mCherry cells. This process was repeated 5 times (5 cell-free passages of the viruses). The expression of GFP on days 4 and 8 of each passage was assessed by a flow cytometry analysis. The copy number of GFP and *env* after each passage was determined by qPCR, as described above.

### *In vivo* experiments.

The *in vivo* experiments were approved by the Mayo Clinic’s Institutional Animal Care and Use Committee. For the evaluation of efficacy of oFV and oFV-GFP in the orthotopic ovarian cancer metastases model, 6-week-old athymic nude mice from the vendor Taconic were intraperitoneally implanted with 2.5 × 10^6^ SKOV-Fluc cells (stably expressing firefly luciferase) (62). Seven days postimplantation, the mice were intraperitoneally injected with a single dose of 10^7^ IU of oFV or oFV-GFP in 200 μl in PBS. The tumor burden was assessed once a week by bioluminescence imaging with IVIS Lumina X5 imaging system after an intraperitoneal injection of luciferin (20 mg/ml). Mice were weighted three times a week. The mice were followed for up to 60 days after infection unless they reached endpoint conditions based on ascites formation or body scoring condition and were euthanized. Ten animals per group were used.

Tumors from PBS- and oFV-GFP-treated mice were harvested at the time of euthanasia and divided into two parts: one-half was explanted by mincing, 1 h incubation with type III collagenase (Stem Cell Technologies), and culture in McCoy's 5A (modified) medium supplemented with 10% FBS and 1% streptomycin/penicillin; the other half of the tumor was embedded in OCT and snap-frozen for cryosectioning and immunohistochemistry. Genomic DNA was isolated from the tumor explants with the DNeasy blood and tissue kits (Qiagen). To detect the presence of the oFV provirus in those tissues, a PCR was run using primers binding within *env* of oFV (forward, GGATGGACCTCCAAACAAAT; reverse, AACCCAATTTCCCAAGCCGT). To detect the transgene, a PCR was run using primers binding to the remaining sequences of *bel2* upstream and downstream of the transgene insertion site (forward, TGTCAGGAGGACCCTTCTGG; reverse, CTGGAGTATTTGGGTAGTGA).

### Immunohistochemistry.

Upon euthanasia, half of each tumor was embedded in OCT and snap-frozen on dry ice. Tumors were then sectioned, fixed in 4% PFA, and incubated overnight at 4°C with primary anti-GFP antibody at 1:250 dilution (rabbit, polyclonal, Abcam). Then, the sections were stained with anti-rabbit secondary antibodies conjugated with Alexa 488 (1:2,000 dilution). The sections were imaged using a Zeiss LSM 510 confocal microscope and analyzed with Zen (Zeiss) software.

### Statistical analysis.

All statistical analyses were done using GraphPad Prism 8, with tests specified in figure legends and alpha = 0.05.

### Diagrams.

Diagrams were created with https://www.BioRender.com.
